# Supplementary material for: Effects of the implementation of the HIV Treat All guidelines on key ART treatment outcomes in Namibia
Source: PLoS One. 2020 Dec 28;15(12):e0243749. doi: 10.1371/journal.pone.0243749 (PMC7769455; doi:10.1371/journal.pone.0243749)
Supplement: Attachment — Submitted filename: Response to Reviewers.docx [file pone.0243749.s001.docx]

**Revised Manuscript Response to Reviewers:**

1. Please ensure that your manuscript meets PLOS ONE's style requirements, including those for file naming. The PLOS ONE style templates can be found at

<https://journals.plos.org/plosone/s/file?id=wjVg/PLOSOne_formatting_sample_main_body.pdf> and

<https://journals.plos.org/plosone/s/file?id=ba62/PLOSOne_formatting_sample_title_authors_affiliations.pdf>

***Response: We have revised the manuscript to ensure all style guidelines are met.***

2. In ethics statement in the manuscript and in the online submission form, please provide additional information about the patient records used in your retrospective study. Specifically, please ensure that you have discussed whether all data were fully anonymized before you accessed them and/or whether the IRB or ethics committee waived the requirement for informed consent. If patients provided informed written consent to have data from their medical records used in research, please include this information.

***Response: Included.***

3. Thank you for stating the following in the Financial Disclosure section:

"This work was supported by Project SOAR (Cooperative agreement AID-OAA-A-14-00060), made possible by the generous support of the American people through the United States President’s Emergency Plan for AIDS Relief (PEPFAR) and United States Agency for International Development (USAID). The contents of this paper are the sole responsibility of the authors and do not necessarily reflect the views of PEPFAR, USAID, or the United States Government. "

We note that one or more of the authors are employed by a commercial company: Survey Warehouse.

3.1. Please provide an amended Funding Statement declaring this commercial affiliation, as well as a statement regarding the Role of Funders in your study. If the funding organization did not play a role in the study design, data collection and analysis, decision to publish, or preparation of the manuscript and only provided financial support in the form of authors' salaries and/or research materials, please review your statements relating to the author contributions, and ensure you have specifically and accurately indicated the role(s) that these authors had in your study. You can update author roles in the Author Contributions section of the online submission form.

Please also include the following statement within your amended Funding Statement.

“The funder provided support in the form of salaries for authors [insert relevant initials], but did not have any additional role in the study design, data collection and analysis, decision to publish, or preparation of the manuscript. The specific roles of these authors are articulated in the ‘author contributions’ section.”

If your commercial affiliation did play a role in your study, please state and explain this role within your updated Funding Statement.

***Responses: We have included this and would like to confirm that the funder has never paid direct salaries to authors for working on this manuscript.***

3.2. Please also provide an updated Competing Interests Statement declaring this commercial affiliation along with any other relevant declarations relating to employment, consultancy, patents, products in development, or marketed products, etc.

Within your Competing Interests Statement, please confirm that this commercial affiliation does not alter your adherence to all PLOS ONE policies on sharing data and materials by including the following statement: "This does not alter our adherence to  PLOS ONE policies on sharing data and materials.” (as detailed online in our guide for authors http://journals.plos.org/plosone/s/competing-interests). If this adherence statement is not accurate and there are restrictions on sharing of data and/or materials, please state these. Please note that we cannot proceed with consideration of your article until this information has been declared.

Please include both an updated Funding Statement and Competing Interests Statement in your cover letter. We will change the online submission form on your behalf.

Please know it is PLOS ONE policy for corresponding authors to declare, on behalf of all authors, all potential competing interests for the purposes of transparency. PLOS defines a competing interest as anything that interferes with, or could reasonably be perceived as interfering with, the full and objective presentation, peer review, editorial decision-making, or publication of research or non-research articles submitted to one of the journals. Competing interests can be financial or non-financial, professional, or personal. Competing interests can arise in relationship to an organization or another person. Please follow this link to our website for more details on competing interests: http://journals.plos.org/plosone/s/competing-interests

***Response: We have included this in the updated cover letter.***

4. We note that you have indicated that data from this study are available upon request. PLOS only allows data to be available upon request if there are legal or ethical restrictions on sharing data publicly. For information on unacceptable data access restrictions, please see <http://journals.plos.org/plosone/s/data-availability#loc-unacceptable-data-access-restrictions>.

In your revised cover letter, please address the following prompts:

a) If there are ethical or legal restrictions on sharing a de-identified data set, please explain them in detail (e.g., data contain potentially identifying or sensitive patient information) and who has imposed them (e.g., an ethics committee). Please also provide contact information for a data access committee, ethics committee, or other institutional body to which data requests may be sent.

b) If there are no restrictions, please upload the minimal anonymized data set necessary to replicate your study findings as either Supporting Information files or to a stable, public repository and provide us with the relevant URLs, DOIs, or accession numbers. Please see <http://www.bmj.com/content/340/bmj.c181.long> for guidelines on how to de-identify and prepare clinical data for publication. For a list of acceptable repositories, please see <http://journals.plos.org/plosone/s/data-availability#loc-recommended-repositories>.

We will update your Data Availability statement on your behalf to reflect the information you provide.

5. We note you have included a table to which you do not refer in the text of your manuscript. Please ensure that you refer to Table 4 in your text; if accepted, production will need this reference to link the reader to the Table.

***Response: We have included a reference to Table 4 in the text.***

**Review Comments to the Author**

Reviewer #1: Reviewer Comments for Manuscript PONE-D-20-12826

General Comments:
The paper addresses and important topic and gives data on what is happening since Test and Treat was implemented. The flow is okay but methods need more detail on what was done and the different outcomes, how they were defined and how they were measured.

***Response: Thank you for your thorough review and useful feedback. We have addressed your comments point by point below:***

Introduction
1. Line 47, may give a clearer picture of Namibia if you give some data on the state of the HIV epidemic in sub-Saharan Africa (SSA) and how the situation in Namibia compares with SSA.

***Response: This has been added and changes are reflected in the tracked version.***

2. Lines 58, 79 introduce times for WHO updated guidelines. Did health facilities go on to implement the 2015 guidance on treating as soon as possible after diagnosis or was nothing done until the 2017 guidelines which gave a more specific time of 7 days. We are missing some information for the period after the 2015 guidelines.

***Response:***

***No specific adoption was made by the Namibian government until April 1, 2017. Detail of the government response is provided in the next paragraph (line 80-94).***

Methods: The methods section needs more clarity on what exactly is being measured as the outcome(s).

1. Study design: You used mixed methods, a retrospective record review and exit interviews. Discuss your study methods again with the team.

***Response: Thank you. Mixed method and exit interviews have been added to the text.***

2. Study sites: You mention northern Namibia and yet this is data to inform the government. Where there no UTAP sites in other regions? Is the population distributed largely in the north and therefore representative of the country? Please justify the choice of only the northern region for data meant to inform the country.

***Response: This has been revised. Please note that the HIV epidemic is concentrated in Northern Namibia and 75% of patients and ART facilities are in 8 Northern districts included in this study.***

***Because this is implementation science research responding to the government need, site selection was determined after careful discussion with MoHSS, USAID and IntraHealth. We also used routine ART service records and monthly client volume data to guide our site selection and ensure geographic representation. Ultimately, six central district hospitals (defined as centralized sites) and four community-based health centers (defined as decentralized sites) from six districts in northern Namibia (Andara, Nyangana, Oshikuku, Onandjokwe, Tsumeb, and Omuthiya). Two of the 8 districts were excluded because the government had another research study on-going at that time.***

3. Line 113 &114: Add the year to the 1st April dates make it clearer which period you are referring to.

***Response: Thank you. The year 2017 has been added.***

4. Line 116 & 117: LTFU is one of the study outcomes, why did you exclude those who were lost after 90 days. Give a justification for this exclusion otherwise it creates bias in estimating the study outcome.

***Response: We followed each cohort of ART patients for 12 months (retrospectively), aiming to understand what happened to them during a period of 12 months. Those who were lost before/ at the beginning of the respective review period were excluded. For example, if we were reviewing and gathering data for the period between April 1, 2016 to April 1, 2017; those who lost before April 1, 2016 were excluded). Including these records would have biased the outcomes.***

***Please also refer to Table 1 to better visualize the time period each data set captured.***

5. Line 132: How did the clients record time that they self-reported? Was there a reliable measure or was it just their perception of whether they waited too long or not?

***Response: We measured wait time at the end of a client visit via exit interview. Participants were asked to report waiting time, including: 1) wait time from arrival at facility to meeting intake nurse; 2) then wait time to see a doctor/ nurse; 3) wait time at the laboratory; and 4) wait time to get a refill. We have added this information to the methods.***

6. Lines 133-138: These give an idea of how satisfaction with service quality was measured. Similarly give a definition for waiting time e.g. cumulative time waiting at certain selected service points like counselling room, lab to give a sample for VL, pharmacy etc. or is it time from arrival at the facility to exit regardless of what happens during that time including clients staying around to wait for their friends?

***Response: Please see #5. Definitions have been added.***

7. Line 140: “New ART clients” and established ART clients are not study outcomes, remove them from the table and replace with “ART initiation” and missed appointment for > 30 days”.

***Response: Thank you. We have revised the subheading of this paragraph and table 1 to include types of ART patients.***

8. Line 145, I don’t think you are able to assess changes over time i.e. throughout the 12 month follow up. You have only 2 measurements, baseline and end line measurements.

***Response: We had two separate data sets representing two time-points (baseline and endline). We were able to assess differences in key outcomes between baseline and endline. In light of your comment, we have revised it to read as: “We conducted bivariate analyses including chi-squared and Fisher’s exact tests to assess differences between baseline and endline and differences between the two service modalities.***

9. Line 146: Mention the non-parametric test you used.

***Response: This has been added: We used median test of equality.***

10. Data management: Give details of data collection tools used (paper vs electronic), the software used for data entry and how validation and query resolution were done.

***Response: Thank you. We made a separate sub-heading for data management. We used tablet-based data collection tools developed using the SurveyCTO electronic data collection platform. Data quality checks were built-in the questionnaires. All research assistants/ interviewers went through a 3-day intensive data collection and ethical training and a 2-day field-testing and practice of the data capture tools.***

Results
1. Lines 158 and 159: The concept of rounds 1 and 2 are introduced for the first time here. These are implied in the section of record extraction (line 108). Go back to methods section and clarify if clients included in round 1 were not included in round 2 and how this was mitigated or was it the same clients having a before and after measurement so that you compared study outcomes at two time points rather than comparing individuals included in the two rounds. Make this clearer to the reader in methods.

***Response:***

1) ***Definition of Round 1 and Round 2 have been included in Table 1. We also included information on the time period each data set contained.***
2) ***We randomly selected medical records of ART clients at two different time points – one year a part. Each data round included retrospective information of clients for a period of 12 months. Clients included in Round 1 could be included again in Round 2. We have added this to the method section. (Please note that this is not a single cohort (we had data of two cohorts) and we did not follow clients prospectively).***

2. Lines 164, 165: The text does not match the data, please correct the statement to show that a higher proportion of clients started ART at stage 2 compared to stage 1 (74% vs 61%; p=0.001).

***Response: We have reviewed this carefully and our text were correct. We presented data in the way that shows whether two rounds of data are comparable. At endline, more clients started ART at Stage 1 (74% compared to 61%).***

3. Lines 166, 167: It is not clear which round had the smaller proportion of established clients!

***Response: Please refer to the column percentages. “Before treat all” data included more established clients (53.6% vs 44.4%).***

4. Table 1: You have used “Before treat All” and “After treat All”. How do these differ from round 1 and round 2? If these are different, please go back to the methods and make it clear what each refers to in terms of periods of the year/ which year. If there are the same, choose one term for consistency either use “rounds” or “before/ after treat All”. It gets confusing as one reads the paper.

***Response: Thank you. As responded above, Round 1 is before treat all; Round 2 is after treat all and both terms are defined in the text.***

5. Lines 175, 176: This is the definition for ART initiation that needs to be included in the study outcomes section of methods so that you do not have to repeat it in subsequent sections of the paper.

***Response: Thank you. We have added the definition of this outcome in Table 1.***

6. Lines 207-211: It is not clear in methods how clients assessed this time. Clarify this in the methods.

***Response: Thank you. We have added this to the methods.***

7. Line 222: What does marginally fewer mean?

***Response: Thank you. We have removed the word “marginally”.***

8. Lines 217-218: Making a diagnosis is something one gets by training and experience. How are the clients able to judge that the diagnosis is not right? It could be implied from the other assessments e.g. health workers do not listen enough, or do not check everything while treating them. These other assessments of patient satisfaction are ok, the one for whether diagnosis is correct should be left out since the p-value does not even show statistical significance.

***Response: We measured perceptions of service quality. We agree with your assessment and that this is not a perfect way for measuring service quality and that is why we used multiple items from a validated tool, and this type of perception can serve as a proxy of service quality.***

Discussion
1. Line 237: Post implementation is another term added. Be consistent with terms used in the text and tables.

***Response: We have removed this term and used “after the implementation of treat all”.***

2. Lines 237-239: These are results, take them to the results section and use the discussion section to “discuss the results”.

***Response: Table 5 has been removed and text has been added to further discuss the decentration process in Namibia***.

3. Lines 248-250: Include the procedures that were done in the UTAP facilities during the time of ART initiation and the days/ weeks after. Did they just let clients go or scheduled follow up counselling sessions e.g. for those who initiated same day treatment. Bringing this information out helps discussion of the results on retention.
4. Line 263: Table 5 should be in the results section.

***Response: Providers followed the national HIV treatment protocol. All patients were counseled before giving ARV drugs, and all patients are scheduled to be back to the facility one month after ART initiation. This has been added.***

Study Limitations
1. Lines 283-285: ART facilities in most countries have data during the time when ART was initiated using CD4 counts. Were these records not available in Namibia from 2010-2014 so that this could be a comparison group?

***Response: What we aimed to say in this paragraph was that due to rapid transition into treat all, it is not ethical or possible to establish a randomized control trial or having a comparison group. Having CD4 would not help or affect our study design, which aimed to investigate the effect of the 2017 Treat All guideline implementation.***

2. Line 288: Recall bias may not arise during records review but more the issues to do with program data not suiting the study purposes so that you end up with missing data on key variables. Also transcribing data from program data bases or tools to the study tools may have led to errors in data collection.

***Response: Thank you. We have added potential error due to recording from the patient care booklets to the data capture tool (electronic data collection).***

3. What proportion of data was missing? Some proportions may be too small that they do not have an effect, state the proportion that was missing only if it is large enough to reduce your study power to detect some associations.

***Response: Thank you. Missing data was not an issue in our client exit interview (under 1%). In our health record reviews, missing data on key outcomes was also not an issue (under 2%). Please note that in our result table, sample size for each key variable was presented and all of our analyses were run on complete data. Due to having a large sample size, we did not face statistical power challenge in our analysis.***

Conclusion
1. Lines 304-305: You could take this to limitations if short follow up time had an effect on your study results. It is not a conclusion.

***Response: Thank you. We agree. However, the conclusion would have been too strong without mentioning that it was only for one year after treat all implementation.***

2. Focus your conclusion on results that you have on ART initiation and high LTFU after implementing new guidelines. Recommendations should come from these.

**Response: Thank you. Key results and recommendations are included.**

3. Lines 308-312: Your study did was not done to demonstrated the feasibility of using existing data to measure key clinical outcomes while strengthening local organizations on collecting and using routine data. It doesn’t seem to fit well in the conclusion section.

***Response: During the conceptualization phase of this study, the government of Namibia as well as donor and implementer were highly interested in knowing if using routine data was feasible. Our study found that this is feasible (as shown in our analysis). During this study, we also trained the local research partner in collecting routine data and analyzing routine data to inform decision making. As routine data is increasingly important, we wanted to stress this in our conclusion.***

Additional reading
You can read through this article that focused on LTFU during “test and treat”. Definition used for T&T was before the 2017 guidelines.

***Response: Thank you.***

Reviewer #2:

I enjoyed reviewing this manuscript and learning more about the implementation of Test and Start in Namibia. The successes offer important insights as other Sub-Saharan African countries seek to scale-up treatment as prevention. The paper also offers methodological insights, through the use of retrospective data, as a randomized design with a control arm is not ethically or practically feasible. I just have a few minor questions and suggestions for the authors.

***Response: Thank you. We have added.***

- In both the introduction and the discussion, I think the authors could say a bit more about how the roll-out of test and start has gone in other Sub-Saharan African (SSA) countries (particularly around the impacts on the health system). Brault, et al. 2020 in Current HIV/AIDS Reports provides a number of references that might be salient. Publications from the MaxART study in Swaziland/eSwatini describe some similar challenges with implementation of test and start in public facilities.

***Response: Thank you for your feedback. We have added some information about the treat-all rollout in Sub-Saharan Africa. Please note that we aim to look at the context of a national rollout of this guideline, which is quite different from a couple of research studies funded by NIH or PEPFAR (i.e. SEARCH or PopART studies). At the time of this study, Botswana, Eswatini, Malawi, and Namibia and South Africa are among the few first countries rolling out treat-all at the national level. We have also included some findings from the MaxART study.***

- More information is needed on the site selection—why only sites in northern Namibia? Also, what was the total number of eligible sites?

***Response: We have provided more information in responding to review #1.***

***Please note that the HIV epidemic is concentrated in Northern Namibia and 75% of patients and ART facilities are in 8 Northern districts included in this study.***

***Because this is an implementation science research in responding to the government need, site selection was determined after careful discussion with MoHSS, USAID and IntraHealth. We also used routine ART service records and monthly client volume data to guide our site selection and ensure geographic representation.***

- It would also be helpful to understand a bit more about the communities/catchment areas of the health facilities. For instance, in and out-migration has been described as a significant factor influencing LTFU and attainment of 90-90-90 goals (here again, see either Brault, et al. 2019 in JAIDS or Brault, et al. 2020). Is this an issue in these areas?

***Response: Thank you. We agreed and overestimating LTFU using routine data (due to silent transfer or migration) is an issue that has been discussed in literature. Migration would occur but there were no observed differences in migration patterns or noticeable ways of migrations during the year before and the year after the implementation of treat all.***

- Testing data (the first 90) is not mentioned at all in the paper. Is testing not an issue in Namibia? If so, this should be explained. If Namibia (like many other SSA countries) continues to struggle with adequately identifying and testing difficult-to-reach populations, then why was this not explored in the present study?

**Response: It was not feasible in our study because we used routine data from ART facilities/ not data from testing sites. HIV testing was done mainly in the community (outreach, provider initiated, patient initiated) at multiple testing sites. Then, positive clients were referred to treatment facilities. We only had information on the date a person was diagnosed. Testing data were not linked to treatment data.**

- The placement of Namibia’s service decentralization timeline in the discussion feels a little out of place. I think this information should be placed in either the introduction or early in the methods.

***Response: Thank you. We agree and have removed the table out of the discussion section. More text has been added.***

- The authors mention the dual challenges of LTFU and lack of facility staff and/or community health workers who can assess client readiness and support new clients. Do they have any recommendations or are there any plans in Namibia to address these issues?

**Response: We have presented the findings and recommendations to the government through a research symposium and they are well taken by the government. At the time of the study, we were not aware of any long-term plans, except task shifting that have been included in the discussion (use of nurse and pharmacies to imitate ART).**

- The authors acknowledge that key limitations of their study are related to time and budget constraints that in turn influenced the design. This has been a challenge for many of the other studies of test and start. Do the authors recommend using a retrospective design to combat these issues, or do they have any recommendations for how to go about this differently in the future?

***Response: Thank you for your feedback. There are a couple of reasons affecting our design; one of which was the challenge in collecting enough newly diagnosed patients for a prospective cohort study because Namibia has a small population of just 2.5 million. In the context of a rapidly evolving treatment strategies, it is important to produce results to the government within a short timeframe and a mixed method implementation research like this one was suitable. Whether a prospective design should be used depends on the research questions. For a research question like this one and in the Namibian context, we would not recommend a prospective cohort.***
