# Supplementary material for: Effects of the implementation of the HIV Treat All guidelines on key ART treatment outcomes in Namibia
Source: PLoS One. 2020 Dec 28;15(12):e0243749. doi: 10.1371/journal.pone.0243749 (PMC7769455; doi:10.1371/journal.pone.0243749)
Supplement: Attachment — Submitted filename: Response to Reviewers_R2.docx [file pone.0243749.s002.docx]

November 14, 2020

Dear the Editorial Office of PLOS ONE,

Please accept our responses to the three (3) minor comments raised by the reviewers for the manuscript entitled *“Effects of the implementation of the HIV Treat All guidelines on key ART treatment outcomes in Namibia”.*

We wish to thank the two reviewers for their thorough reviews and helpful feedback.

Best regards,

Lung Vu, MD, PhD

Senior Health Specialist/Data Scientist

Health, Nutrition and Population

T [+1 (202) 509 3748](tel:+1%20(202)%20730%206898)

E [lvu8@worldbank.org](mailto:lvu8@worldbank.org)

W [www.worldbank.org/en/topic/health](http://www.worldbank.org/en/topic/health)

**Reviewer #1:**

General Comments: Reviewer comments have been addressed; the paper is accepted pending a minor correction as mentioned below.

Minor comment: Lines 129 & 130: Add the year to the 1st April dates to make it clearer which period you are referring to.

***Response: The date of April 1 refers to the start date for both the pre-Treat All cohort (April 1, 2016) and the post-Treat All cohort (April 1, 2017). We have revised the text in lines 126 to 132 to provide better clarity about timing.***

**Reviewer #2**

1) The authors have mostly addressed the comments in the previous manuscript. However, they stated that they incorporated findings from the MaxART study in the revised manuscript, but I did not see any manuscripts from MaxART in the references. I think that the authors could do a better job linking this manuscript to more recent studies and reviews of treatment as prevention.

***This link has been clarified at lines 259-260. The article entitled “Experiences from the MaxART Early Access to ART for All (EAAA) Trial in Eswatini” is now included in our citation list (citation #18 in the clean version).***

2) In addition, I disagree with the description of the study as "mixed methods", as no qualitative data was collected or analyzed. The interviews appeared to have been structured and quantitative. If qualitative data was collected, then please include details on the qualitative data collection methods (including interview guide as an appendix), and qualitative analysis methods (grounded theory, framework, other approaches to coding and analysis).

***Response: We have removed reference to the mixed-method design as this article does not systematically draw from the qualitative interviews conducted for this study. The text on lines 109-111 now reads: “This is implementation science study uses retrospective, observational data extracted from longitudinal clinical health record data and collected through exit interviews from a random sample of HIV care and treatment clients at health facilities in Namibia at 2 separate timepoints.”***

3) Finally, in responding to reviewers’ comments, it would be helpful to provide more details, such as the page and line numbers, where the changes were made.

***Response: We appreciate this feedback and regret the oversight. We have included line numbers with the changes noted above and will be sure to include them in future revision memos to assist reviewers.***
